# Compliance With Parathyroid Hormone Analog Medications at a Single Osteoporosis Treatment Center

**DOI:** 10.7759/cureus.82773

**Published:** 2025-04-22

**Authors:** Andrea H Johnson, Jane C Brennan, Daniel Boudani, Lauren Chapa, Gerald Kidd, Justin J Turcotte, Christina Morganti

**Affiliations:** 1 Orthopedics, Anne Arundel Medical Center, Annapolis, USA; 2 Orthopedic Research, Anne Arundel Medical Center, Annapolis, USA; 3 Orthopedic and Surgical Research, Anne Arundel Medical Center, Annapolis, USA; 4 Orthopedic Surgery, Anne Arundel Medical Center, Annapolis, USA

**Keywords:** abaloparatide, medication compliance, osteoporosis, parathyroid hormone analog, teriparatide

## Abstract

Background

Osteoporosis is the most common metabolic bone disease and is more common in female patients and the elderly. The costs associated with osteoporotic fractures present a significant burden on the healthcare system. While effective pharmacologic therapy exists, treatment initiation and compliance remain suboptimal, leading to increased morbidity. This study evaluated compliance with parathyroid hormone analogs and identified reasons for non-compliance.

Methods

This was a retrospective study of patients prescribed parathyroid hormone analogs from a single osteoporosis clinic from 2017 to 2022. Of the 100 patients included, 42 were prescribed teriparatide and 58 were prescribed abaloparatide. The primary outcomes of interest were continued compliance with medication therapy and reasons for non-compliance. The secondary outcome of interest was fracture rate. Univariate analyses were performed to identify differences between the two groups, and a Kaplan-Meier curve was generated to show overall compliance.

Results

The average treatment duration was 15.5 months (95% CI: 13.5-17.4 months); 54 (54%) patients completed the full 24-month medication course. Abaloparatide patients had higher rates of non-compliance than teriparatide patients (33 (56.9%) vs. 13 (31.0%); p=0.018) and had a significantly shorter time to non-compliance (4.1 ± 4.6 vs. 8.8 ± 6.4 months; p=0.027). There was a significant difference in reason for non-compliance between early (≤3 months) and late non-compliant patients (p=0.001). About 19 (79.2%) patients were non-compliant early because of side effects, while 10 (45.5%) patients were non-compliant late because of financial reasons. There were no differences in fracture rates between the two groups.

Conclusion

More than half of the patients in this study completed the full two-year course of treatment, and the average duration of treatment was over 15 months. The overall fracture rate during and after treatment was low. Financial concerns and side effects were the most common reasons for non-compliance. An additional study would be needed to evaluate methods for improving patient compliance with parathyroid hormone analogs.

## Introduction

Osteoporosis is the most common metabolic bone disease in the world and is characterized by low bone mineral density, disrupted bone microarchitecture, decreased bone strength, and fracture [1]. It is a significant public health issue, with the National Osteoporosis Foundation estimating that 43.4 million Americans have low bone mass and an additional 10.2 million have osteoporosis, contributing to more than two million osteoporosis-related fractures annually [2]. Osteoporosis is more common in female patients and the elderly, and the costs associated with fracture care place a significant and increasing burden on the health system [3]. In a study of women aged 55 and older, osteoporotic fractures accounted for more hospitalizations than myocardial infarction, stroke, or breast cancer [4]. Despite the significant issues that osteoporosis presents, it remains a very undertreated condition even after osteoporotic fracture [5]. In patients who initiate osteoporosis treatment, treatment compliance remains a significant issue [6-10].

There are a number of classes of medications to treat osteoporosis, along with a variety of administration timings and routes [1,2]. Parathyroid hormone analogs, including teriparatide and abaloparatide, are highly efficacious in patients who are at high risk for fracture [1,11,12]. The recombinant parathyroid hormone stimulates bone formation and may improve the bone microarchitecture, though use of these medications is typically limited to a two-year course of treatment [1,2]. Typically, a course of these medications is followed by initiating an antiresorptive agent in order to prevent rapid bone loss, which is associated with medication discontinuation [11]. Several studies have shown improved compliance with these medications compared with other classes of osteoporosis medications despite the fact that they require a daily self-administered injection [6,10,13]. The purpose of this study was to evaluate patient compliance with teriparatide and abaloparatide over the two-year course of treatment in a single osteoporosis treatment clinic and to evaluate the differences between compliant and non-compliant patients as well as the reasons for noncompliance.

## Materials and methods

This was a retrospective observational cohort study of patients referred to Anne Arundel Medical Center, Annapolis, USA, from December 2017 to December 2022. This was a single-institutional study using patient data obtained from patient records in the electronic health record (EHR). The purpose of this study was to identify treatment patterns and compliance for patients referred to a dedicated osteoporosis clinic. 

Study population

A total of 123 patients prescribed teriparatide or abaloparatide from December 2017 to December 2022 at a dedicated community-based osteoporosis treatment clinic were included, with 23 of these patients ultimately lost to follow-up. Of the 100 patients that remained, 42 were prescribed teriparatide and 58 were prescribed abaloparatide.

Outcomes

The primary outcomes of interest were continued compliance with prescribed osteoporosis medication, reasons for non-compliance with medication, and early/late non-compliance. Non-compliance was defined as stopping the medication permanently. The secondary outcome of interest was fracture rate.

Independent variables

The independent variables of interest were age, sex, race, body mass index (BMI), insurance, smoking status, history of fragility fracture, use of assistive device, cognitive dysfunction, current regular exercise, daily calcium supplement, daily vitamin D supplement, any previous bone medications, chronic kidney disease, liver disease, diabetes, history of cancer, thyroid disease, autoimmune disease, and family history of osteoporosis.

Statistical analysis

A Kaplan-Meier survival curve was generated to show overall compliance. Patients were then divided into compliant or non-compliant based on their compliance with their prescribed medication. Differences in patient characteristics and history between compliant and non-compliant patients were compared using two-sided independent samples t-tests for continuous measures and chi-squared tests for categorical measures. Differences in rates of non-compliance, time to non-compliance, and reason for non-compliance were compared between patients prescribed teriparatide and those prescribed abaloparatide using two-sided independent samples t-tests and chi-squared tests for categorical measures. Non-compliant patients were divided into early (less than or equal to three months) and late (greater than three months) non-compliance based on when they stopped taking their medication. Differences in patient characteristics and history, rates of non-compliance, time to non-compliance, and reason for non-compliance were compared between early and late non-compliant patients using two-sided independent samples t-tests and chi-squared tests for categorical measures. Finally, differences in fracture rates between compliant and non-compliant patients, early and late non-compliant patients, and teriparatide and abaloparatide patients were compared using chi-squared tests. All statistical analyses were performed using RStudio v4.2.2 (Posit, Boston, USA). Statistical significance was assessed at p<0.05. 

## Results

**Figure 1:**
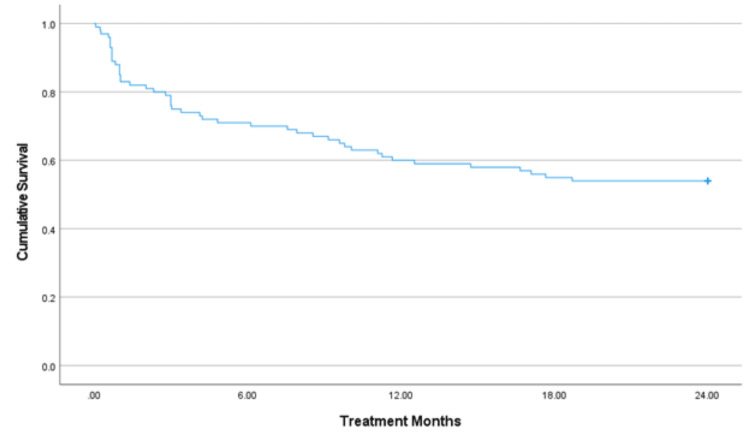
Medication compliance analyzed using the Kaplan-Meir survival curve The average treatment duration was 15.5 months (95% CI: 13.5 to 17.4 months). At 12 months, approximately 60% of patients remained on treatment. In total, 54% of patients completed the full 24-month medication regimen.

The average duration of treatment was 15.5 months (95% CI: 13.5 to 17.4 months). At 12 months, approximately 60% of patients remained on treatment. Overall, 54% of patients completed the full 24-month medication course (Figure 1).

 There were no differences in patient characteristics or history between compliant and non-compliant patients (Table 1).

**Table 1:** Patient history and characteristics by medication compliance All data presented as n (%) or mean ± SD. * indicates Fisher’s exact test; t-score indicates two-sided t-test; X^2^ indicates chi-squared test

Patient Characteristic	Compliant (n=54)	Non-compliant (n=46)	Test Statistic	P-value
Age, years	64.3 ± 8.8	63.6 ± 5.6	t-score = 0.447	0.656
Body mass index, kg/m^2^	24.8 ± 6.0	26.1 ± 6.8	t-score = -0.977	0.331
Sex				0.060*
Male	5 (9.3)	0 (0)		
Female	49 (90.7)	46 (100)		
Non-White race	0 (0)	2 (4.3)		0.198*
Insurance				
Private	34 (63.0)	22 (47.8)	X^2^ = 1.736	0.188
Medicare	20 (37.0)	23 (50.0)	X^2^ = 1.215	0.270
Medicaid	0 (0)	0 (0)		1*
Uninsured	0 (0)	1 (2.2)		0.460*
Smoking status				0.526*
Current	4 (7.4)	2 (4.3)		
Former	17 (31.5)	11 (23.9)		
Never	33 (61.1)	33 (71.7)		
History of fragility fracture	41 (75.9)	35 (76.1)	X^2^ = 0	1
Any assistive device	5 (9.3)	6 (13.0)	X^2^ = 0.080	0.296
Cognitive dysfunction	0 (0)	1 (2.2)		0.460*
Current regular exercise	40 (74.1)	40 (87.0)	X^2^ = 1.834	0.176
Calcium supplement	34 (63.0)	23 (50.0)	X^2^ = 1.215	0.270
Vitamin D supplement	43 (79.6)	35 (76.1)	X^2^ = 0.034	0.854
Previous bone medication	19 (35.2)	20 (43.5)	X^2^ = 0.419	0.521
Chronic kidney disease	1 (1.9)	1 (2.2)		1*
Liver disease	1 (1.9)	1 (2.2)		1*
Diabetes				0.200*
Diet controlled	0 (0)	1 (2.2)		
Medication controlled	2 (3.7)	0 (0)		
Other	0 (0)	2 (4.3)		
None	52 (96.3)	43 (93.5)		
History of cancer	13 (24.1)	8 (17.4)	X^2^ = 0.327	0.568
Thyroid disease	11 (20.4)	5 (10.9)	X^2^ = 1.036	0.309
Autoimmune disease				0.811*
Rheumatoid arthritis	3 (5.6)	2 (4.3)		
Systemic lupus	1 (1.9)	0 (0)		
Other	3 (5.6)	1 (2.2)		
Family history of osteoporosis	37 (68.5)	26 (56.5)	X^2^ = 1.062	0.303
Total number of visits	8.3 ± 2.2	7.5 ± 3.3	t-score = 1.357	0.179

Abaloparatide patients had higher rates of non-compliance than teriparatide patients (56.9% vs. 31.0%; p=0.018) and had a significantly shorter time to non-compliance (4.1 ± 4.6 months vs. 8.8 ± 6.4; p=0.027). The reasons for non-compliance were different between abaloparatide and teriparatide patients (p<0.001); 69.7% of abaloparatide patients were non-compliant due to side effects, while 69.2% of teriparatide patients were non-compliant due to financial reasons (Table 2).

**Table 2:** Reasons for non-compliance by medication prescribed All data presented as n (%) or mean ± SD. P-value <0.05 in bold. * indicates Fisher’s exact test; t-score indicates two-sided t-test; X^2^ indicates chi-squared test; ** calculated for non-compliant patients only (n=46)

	All Patients (n=100)	Teriparatide (n=42)	Abaloparatide (n=58)	Test Statistic	P-value
Non-compliant	46 (46.0)	13 (31.0)	33 (56.9)	X^2^ = 5.598	0.018
Time to non-compliance (months)**	5.4 ± 5.5	8.8 ± 6.4	4.1 ± 4.6	t-score = 4.060	0.027
Reason for non-compliance**					<0.001*
Financial	14 (30.4)	9 (69.2)	5 (15.2)		
Patient decision	3 (6.5)	2 (15.4)	1 (3.0)		
Provider switched	5 (10.9)	1 (7.7)	4 (12.1)		
Side effects	24 (52.2)	1 (7.7)	23 (69.7)		

Of the 46 non-compliant patients, 52.2% were non-compliant early and 47.8% were non-compliant late. Late non-compliant patients, on average, had a higher BMI than early non-compliant patients (28.9 ± 7.2 vs. 23.5 ± 5.3; p=0.008), and there was a significant difference in reasons for non-compliance between early and late non-compliant patients (p=0.001). About 79.2% of early non-compliant patients were non-compliant because of side effects, while 45.5% of late non-compliant patients were non-compliant because of financial reasons (Table 3).

**Table 3:** Patient characteristics and reasons for non-compliance (early vs. late non-compliance) All data presented as n (%) or mean ± SD. P-value <0.05 in bold. * indicates Fisher’s exact test; t-score indicates two-sided t-test; X^2^ indicates chi-squared test

Patient Characteristic	Early Non-compliance (≤3 months) (n=24)	Late Non-compliance (>3 months) (n=22)	Test Statistic	P-value
Age, years	62.8 ±5.3	64.5 ±5.9	t-score = -0.978	0.334
Body mass index, kg/m^2^	23.5 ± 5.3	28.9 ± 7.2	t-score = -2.816	0.008
Sex				1*
Male	0 (0)	0 (0)		
Female	24 (100)	22 (100)		
Non-White race	2 (8.3)	0 (0)		0.491*
Insurance				
Private	12 (50.0)	10 (45.5)	X^2^ = 0.0002	0.758
Medicare	11 (45.8)	12 (54.4)	X^2^ = 0.087	0.555
Medicaid	0 (0)	0 (0)		1*
Uninsured	1 (4.2)	0 (0)		1*
Smoking status				0.439*
Current	0 (0)	2 (9.1)		
Former	6 (25.0)	5 (22.7)		
Never	18 (75.0)	15 (68.2)		
History of fragility fracture	19 (79.2)	16 (72.7)	X^2^ = 0.027	0.869
Assistive device	3 (12.5)	3 (13.6)		1*
Cognitive dysfunction	1 (4.2)	0 (0)		1*
Current regular exercise	23 (95.8)	17 (77.3)		0.090*
Calcium supplement	14 (58.3)	9 (40.9)	X^2^ = 0.784	0.376
Vitamin D supplement	18 (75.0)	17 (77.3)	X^2^ <0.001	1
Previous bone medication	12 (50.0)	8 (36.4)	X^2^ = 0.402	0.526
Chronic kidney disease	0 (0)	1 (4.5)		0.478*
Liver disease	1 (4.2)	0 (0)		1*
Diabetes mellitus				1*
Diet controlled	1 (4.2)	0 (0)		
Medication controlled	0 (0)	0 (0)		
Other	1 (4.2)	1 (4.5)		
None	22 (91.7)	21 (95.5)		
History of cancer	5 (20.8)	3 (13.6)		0.702*
Thyroid disease	2 (8.3)	3 (13.6)		0.659*
Autoimmune disease				0.223*
Rheumatoid arthritis	0 (0)	2 (9.1)		
Systemic lupus	0 (0)	0 (0)		
Other	1 (4.2)	0 (0)		
Family history of osteoporosis	15 (62.5)	11 (50.0)	X^2^ = 0.310	0.578
Total number of visits	7.0 ± 2.5	8.1 ± 4.0	t-score = -1.146	0.260
Medication			X^2^ = 2.239	0.135
Teriparatide	4 (16.7)	9 (40.9)		
Abaloparatide	20 (83.3)	13 (59.1)		
Reason for non-compliance				<0.001*
Financial	4 (16.7)	10 (45.5)		
Patient decision	0 (0)	3 (13.6)		
Provider switched	1 (4.2)	4 (18.2)		
Side effects	19 (79.2)	5 (22.7)		

There were no differences in fracture rates during/after medication therapy between compliant and non-compliant patients, early and late non-compliant patients, or teriparatide and abaloparatide patients (Table 4).

**Table 4:** Fracture rates during/after medication therapy All data presented as n (%). * indicates Fisher’s exact test; N/A: not applicable

Comparison Group	Fracture Rate	P-value
All patients	9/100 (9.0%)	N/A
Non-compliant	7/46 (15.2%)	0.076*
Compliant	2/54 (3.7%)	
Early non-compliance	4/24 (16.7%)	1.000*
Late non-compliance	3/22 (13.6%)	
Teriparatide	3/42 (7.1%)	0.730*

## Discussion

Over the planned two-year course of treatment for patients treated with abaloparatide or teriparatide, nearly two-thirds of patients were compliant at 12 months and just over half of patients were compliant for the full 24 months. Patients prescribed teriparatide were more likely to stay compliant and complete the course of treatment, although they were more likely to identify financial issues as the reason for non-compliance, while patients prescribed abaloparatide were more likely to identify side effects as the reason for non-compliance. Patients who became non-compliant in the first three months were more likely to identify side effects as the reason for non-compliance, while patients who became non-compliant later on attributed this to financial issues. There were no differences in fracture rates between groups throughout the study, although there was a trend toward increased fracture rates in non-compliant patients.

Rates of compliance with osteoporosis medication can vary widely and are influenced by many factors. Compared with bisphosphonate medications and other antiresorptive agents for osteoporosis treatment, patients prescribed anabolic agents often have better medication compliance [10,14]. Anabolic agents are also typically reserved for patients with osteoporosis at high risk for fracture, unlike other medications that have a wider indication, which may influence compliance due to increased patient motivation [11,15]. A study by Durden et al. found that patients prescribed an injectable osteoporosis medication were more likely to be compliant compared to patients prescribed an oral medication, although the overall rate of compliance with any medication was low at 20-41% over two years [10]. In a large-scale study of medication compliance with osteoporosis medications, Reyes et al. found that the one-year compliance with teriparatide was 58% and dropped to 19% at two years, while patients taking alendronate had 48% compliance at one year and 29% at two years [14]. In a recent study by Gold et al., nearly 65% of patients prescribed abaloparatide completed the course of treatment [6]. In a meta-analysis by Koller et al., patients taking teriparatide had a weighted average compliance of 48% at two years [13]. In comparison, patients in our study had relatively high compliance, with 54% of patients completing the full two-year course of treatment. When comparing patients taking teriparatide with patients taking abaloparatide, patients taking teriparatide were more likely to remain compliant for the duration of treatment. 

It is important to identify reasons for patient non-compliance with osteoporosis medications in order to adequately address this issue. Gastrointestinal (GI) side effects are commonly associated with oral osteoporosis medications, particularly bisphosphates, and these symptoms are often cited as a reason for discontinuation of these medications [8,16]. The anabolic agents currently in the market, including teriparatide and abaloparatide, are injectable medications and do not typically cause high levels of GI symptoms that can be experienced with oral medications [17]. Woo et al. found that GI symptoms were a significant risk factor for early discontinuation of osteoporosis treatment [16]. Common side effects associated with teriparatide and abaloparatide include injection site reactions, palpitations, tachycardia, nausea, headache, dizziness, and extremity pain [18-20]. While this study did not specify the type of side effect, approximately one-quarter of patients in the study attributed medication side effects as the reason for non-compliance. Financial considerations and medication costs can also be important factors to consider. In a study by Deng et al., of nearly 700 patients prescribed a variety of osteoporosis medications, approximately 8% of patients discontinued medication due to financial concerns [9]. In a study looking specifically at abaloparatide compliance, Gold et al. found that 31% of patients discontinued treatment due to financial concerns [6]. Of note, the United States patent for teriparatide expired in 2019, and biosimilar medication became available, which may have affected medication price and industry support (i.e., sample medication availability) [21,22]. Similarly, this study found a similar rate of non-compliance due to financial concerns, with just over 30% of patients reporting that as the reason for non-compliance.

Given that osteoporosis medications are prescribed in order to decrease fracture risk, examining the rate of fractures for patients taking these medications is vital. Burge et al. found that in a large administrative database study, fracture rates overall and specifically vertebral, nonvertebral, and hip fracture rates decreased as adherence to teriparatide use increased [23]. In a meta-analysis by Chen et al., adherence to teriparatide use decreased the rate of all fractures by 28%, decreased the rate of hip fractures by 49%, and decreased the rate of nonvertebral fractures by 26% [12]. In a study by Keshishian et al. of female Medicare enrollees, patients who had low adherence to osteoporosis medication had a 32% increased risk for hip or pelvis fracture and a 34% increased risk for vertebral fracture compared to high adherence patients [24]. Patients in this study who were non-compliant with medication had approximately four times the risk of fracture compared to compliant patients; however, this result did not reach statistical significance.

There are a number of methods that have been investigated in order to increase compliance with osteoporosis medications. A study by Sato et al. found that patients prescribed teriparatide who were also enrolled in a patient support program had higher rates of compliance than those who were not [25]. Cheng et al. found that patients using a mobile health platform were able to effectively increase their knowledge and compliance regarding osteoporosis [26]. van Maren et al. found that patient compliance with teriparatide increased after the introduction of a patient education and support program [27]. While this study did not implement any medication-specific patient support programs, compliant patients had, on average, one additional visit to the osteoporosis clinic than non-compliant patients, although this was not statistically significant.

This study does need to be considered in light of its limitations. It is a retrospective study from a single institution, and the results may not be comparable to a wider patient population. The retrospective nature of the study also introduces inherent selection bias, although we included all patients prescribed abaloparatide or teriparatide through this osteoporosis clinic. Medication selection was determined by patient and provider discussion and relied on several variables, including insurance coverage, sample medication availability, and patient and provider preference. The sample size was relatively small and the fracture rate was low overall, which may leave the study underpowered to detect significant differences between groups. Because we collected data directly from the clinic's medical records, we gathered substantial information regarding treatment patterns and compliance, including reasons for non-compliance, which may provide further insight to improve compliance in the future.

## Conclusions

More than half of the patients prescribed teriparatide and abaloparatide in this study completed the full two-year course of treatment, and the average duration of treatment exceeded 15 months. The overall fracture rate during and after treatment was low. Patients who were prescribed teriparatide and those who became non-compliant within three months of initiating treatment were more likely to cite financial concerns as the reason for non-compliance. Patients who were prescribed abaloparatide and those who became non-compliant more than three months after initiating treatment were more likely to report side effects as the reason for non-compliance. Additional studies are needed to further evaluate factors contributing to non-compliance and methods for improving patient compliance while taking osteoporosis medications. 
